# Functional Analysis of the Unique Cytochrome P450 of the Liver Fluke *Opisthorchis felineus*

**DOI:** 10.1371/journal.pntd.0004258

**Published:** 2015-12-01

**Authors:** Mariya Y. Pakharukova, Valentin A. Vavilin, Banchob Sripa, Thewarach Laha, Paul J. Brindley, Viatcheslav A. Mordvinov

**Affiliations:** 1 Laboratory of Molecular Mechanisms of Pathological Processes, Institute of Cytology and Genetics, Siberian Branch of the Russian Academy of Sciences, Novosibirsk, Russia; 2 Department of Natural Sciences, Novosibirsk State University, Novosibirsk, Russia; 3 Laboratory of Pharmacokinetic and Drugs Metabolism, Institute of Molecular Biology and Biophysics, Siberian Branch of the Russian Academy of Medical Sciences, Novosibirsk, Russia; 4 Tropical Disease Research Laboratory, Liver Fluke and Cholangiocarcinoma Research Center, Department of Pathology, Faculty of Medicine, Khon Kaen University, Khon Kaen, Thailand; 5 Department of Parasitology, Faculty of Medicine, Khon Kaen University, Khon Kaen, Thailand; 6 Department of Microbiology, Immunology and Tropical Medicine, and Research Center for Neglected Diseases of Poverty, School of Medicine & Health Sciences, George Washington University, Washington, D.C., United States of America; University of Nottingham, UNITED KINGDOM

## Abstract

The basic metabolic cytochrome P450 (CYP) system is essential for biotransformation of sterols and xenobiotics including drugs, for synthesis and degradation of signaling molecules in all living organisms. Most eukaryotes including free-living flatworms have numerous paralogues of the CYP gene encoding heme monooxygenases with specific substrate range. Notably, by contrast, the parasitic flatworms have only one CYP gene. The role of this enzyme in the physiology and biochemistry of helminths is not known. The flukes and tapeworms are the etiologic agents of major neglected tropical diseases of humanity. Three helminth infections (*Opisthorchis viverrini*, *Clonorchis sinensis* and *Schistosoma haematobium*) are considered by the International Agency for Research on Cancer (IARC) as definite causes of cancer. We focused our research on the human liver fluke *Opisthorchis felineus*, an emerging source of biliary tract disease including bile duct cancer in Russia and central Europe. The aims of this study were (i) to determine the significance of the CYP activity for the morphology and survival of the liver fluke, (ii) to assess CYP ability to metabolize xenobiotics, and (iii) to localize the CYP activity in *O*. *felineus* tissues. We observed high constitutive expression of CYP mRNA (Real-time PCR) in *O*. *felineus*. This enzyme metabolized xenobiotics selective for mammalian CYP2E1, CYP2B, CYP3A, but not CYP1A, as determined by liquid chromatography and imaging analyses. Tissue localization studies revealed the CYP activity in excretory channels, while suppression of CYP mRNA by RNA interference was accompanied by morphological changes of the excretory system and increased mortality rates of the worms. These results suggest that the CYP function is linked to worm metabolism and detoxification. The findings also suggest that the CYP enzyme is involved in vitally important processes in the organism of parasites and is a potential drug target.

## Author Summary

The basic metabolic system CYP (cytochrome P450) is essential for biotransformation of sterols and xenobiotics, for synthesis and degradation of signaling molecules in all living organisms. Most eukaryotes including free-living flatworms evolved numerous paralogues of the CYP gene. Notably, by contrast, flukes and tapeworms–the etiologic agents of major neglected tropical diseases of humanity, have only one gene. However, the role of P450 in the physiology and biochemistry of helminths is not known. This report presents the first functional study of the CYP enzyme of any of the parasitic flatworms. We focused our research on the food-borne human liver fluke, *Opisthorchis felineus*, an emerging source of biliary tract diseases in Russia, Kazakhstan and central Europe. Here we report that this liver fluke has evolved a highly expressed functional monooxygenase system with broad substrate specificity. Tissue localization studies and suppression of CYP mRNA by RNA interference revealed the CYP function is linked to the excretory system and possibly to metabolism and detoxification. The fluke’s monooxygenase likely is a model for orthologues of the singular CYP of parasitic flatworms at large, where it plays a critical role in the pathogen’s metabolism that contributes to worm survival and drug resistance.

## Introduction

The heme-containing enzymes cytochromes P450 (CYPs) are widely distributed in living organisms from bacteria to mammals [reviewed in Refs. 1, 2–3]. CYP enzymes act as components of a monooxygenase system; they display biological functions ranging from detoxification of environmental pollutants to synthesis and degradation of endogenous signaling molecules [1]. More than 80% of existing prescription drugs undergo the unavoidable step of biotransformation mediated by cytochromes Р450. This step typically limits the speed of the biotransformation process and of excretion of the drugs [1].

Given these roles, CYP are functionally important for survival of invading pathogens. For example, CYP in *Leishmania donovani* is essential for cell growth, infection, ergosterol biosynthesis, and other processes. Parasites with one allele of CYP show impaired growth, lower membrane potential, reduced virulence, and higher sensitivity to drugs [2]. Inactivation of both copies of a CYP gene is lethal for this microbe [2]. In the free-living nematode *Caenorhabditis elegans*, one of the CYP enzymes participates in the synthesis of a steroid hormone necessary for worm development [3], whereas the other CYP enzyme is involved in fatty acids homeostasis [4]. It is known that in fungi, the CYP system is an important pharmacological target because it participates in the synthesis of the cell wall of spores, in the metabolism of membrane sterols, and in production of metabolites with antibacterial properties [1].

It is noteworthy that CYP enzymes may be linked to the synthesis of unique sterol-like metabolites, oxysterols, and catechol-estrogens of specific structure known from trematodes, specifically *Opisthorchis viverrini* and *Schistosoma haematobium* [5, 6]. Oxysterols, which are oxidation products of cholesterol and are generated by enzymatic (cytochromes P450) or non-enzymatic processes [7], can be mutagenic and genotoxic and may possess pro-oxidative and proinflammatory properties that promote carcinogenesis [8–9]. Accordingly, analysis and characterization of the function of CYP of parasites can be anticipated to lead to a deeper understanding of diverse aspects of parasite physiology that contribute to cell survival, drug resistance, and maintenance and evolution of the host-parasite relationship.

Despite importance of the CYP system for the physiology of parasites and other pathogens and for drug detoxification, CYP enzymes of eukaryotic parasites remain poorly understood. It had long been thought that parasitic worms lost CYPs [10]. Recently, monooxygenase transformation of certain drugs into inactive metabolites was demonstrated in flukes [11–12]. The role of this activity in the drug resistance of parasites was also described [11–13]. For example, increased formation of oxidized inactive metabolites of albendazole and triclabendazole in resistant isolates of parasitic flatworms has been reported; furthermore, inhibition of the oxidative metabolism increases sensitivity of the worms to anthelminthic drugs [13]. In addition, a monooxygenase activity was discovered in *S*. *mansoni* and *S*. *haematobium* [14]. These findings indicated the existence of a CYP system in parasitic clades of the phylum Platyhelminthes; however, data are not yet available on either the composition of the CYP system or the function of these enzymes in flatworms.

The liver flukes *O*. *felineus*, *O*. *viverrini*, and *Clonorchis sinensis* of the family Opisthorchiidae (Class Trematoda) cause serious human diseases affecting bile ducts and the gall bladder. The International Agency for Research on Cancer (IARC) recognizes infection with two of these three helminths as a definitive cause of cholangiocarcinoma: the liver flukes *O*. *viverrini* and *C*. *sinensis* [15]. Carcinogenic effects of an infection are also possible for *O*. *felineus*, given the similar signs of infection development and disease course [16]. *O*. *felineus* (Rivolta, 1884) occurred primarily on the territory of the former USSR, but increasingly it is being found in other regions of Europe [see Refs. 17–18]. It is estimated that worldwide, there are 1.6 million cases of opisthorchiasis resulting from infection with *O*. *felineus*. Despite its public health significance, this widespread Eurasian species is one of the most poorly studied human liver flukes.

The ‘CYPome’ of parasitic flatworms, including liver flukes (Opisthorchiidae, Fasciolidae), blood flukes (Schistosomatidae), and cestodes (Taeniidae) contains only one CYP gene [19]. Additionally, flavin monooxygenase genes appeared to be absent. Apparently, in these parasites, the main enzyme that has a monooxygenase activity toward xenobiotics is CYP. We cloned and sequenced CYP cDNA of *O*. *felineus* (GenBank ID: JF920147), and predicted the structure of the protein it encodes. The predicted protein has conserved structure, contains functional domains (characteristic of mammalian microsomal CYP enzymes involved in biotransformation of xenobiotics) and turned out to be most closely related in its structure to the mammalian CYP2 subfamily. Furthermore, this CYP gene exhibits strong expression of mRNA. Nevertheless, little or nothing is known about the functions of the protein.

The aim of the present study was to analyze the functional organization of the metabolic system of cytochromes P450 in *O*. *felineus*, the liver fluke of humans and other fish-eating mammals. In particular, we set out to determine the functional significance of the monooxygenase of *O*. *felineus*, to assess its ability to metabolize xenobiotics, to identify the possible spectrum of substrate specificity of this CYP, evaluate the inducibility of the CYP gene, and to determine the necessity of expression of this gene on the phenotype of the liver fluke.

## Materials and Methods

### Ethics statement

All of the procedures were in compliance with The Code of Ethics of the World Medical Association (Declaration of Helsinki) for animal experiments http://ec.europa.eu/environment/chemicals/lab_animals/legislation_en.htm. The hamsters were kept and treated according to protocols approved by the Committee on the Ethics of Animal Experiments of the Institute of Cytology and Genetics (Permit Number: 25 of 12.12.2014).

### Animals and parasites

Golden Syrian hamsters (*Mesocricetus auratus*) were purchased from the Puschino Animal Facility (Russia) and subsequently bred at the Animal Facility of the Institute of Cytology and Genetics, SB RAS (RFMEFI61914X0005), (Russia). Euthanasia was performed by decapitation, and all efforts were made to minimize suffering.

Metacercariae of *O*. *felineus* were collected from naturally infected fish (*Leuciscus idus*) from the Ob River near the city of Novosibirsk, Western Siberia. Territories where sample collection (fishing) took place were neither conservation areas nor private, nor otherwise protected; hence, no fishing permits were required. The fish species collected are not considered endangered or rare, and the fishing methods complied with the Federal Law N166-F3 of 20.12.2004 (ed. 18.07.2011), "Fishing and conservation of water bio-resources”.

### *In vitro* treatment of liver flukes

Adult liver flukes were recovered from the hepatobiliary tract of hamsters infected three months earlier with 100 metacercariae [20, 21]. Newly excysted metacercariae (NEM) were hatched from metacercariae using incubation with 0.1% trypsin in sterile saline solution at 37°C for 15 min [21]. The parasites were incubated in the RPMI medium supplemented with 1× antibiotic/antimycotic (Sigma–Aldrich, USA) and 1% glucose at 37°C for either 4 or 24 h with one of the xenobiotics. Xenobiotics that were dissolved in dimethyl sulfoxide (DMSO, Sigma–Aldrich) were added in the form of 100× stock solutions to final concentrations of 10 nM 2,3,7,8-tetrachlorodibenzodioxin (TCDD), 50 μM phenobarbital (PB; Fluka, Switzerland), 10 μM dexamethazone (DEX; Sigma–Aldrich), 50 mM ethanol, 0.1 μg/mL praziquantel (PZQ; Bayer, Germany), or 40 μM ketoconazole (Zdorovie, Russia). Cholesterol (Sigma–Aldrich) was dissolved in 96% ethanol at the concentration of 10 mg/mL to prepare a 2000× stock solution. Bile acids (Sigma–Aldrich) were dissolved in water to prepare a stock solution 400 mg/mL and were added to the medium as a 100× solution. Hemoglobin (Sigma–Aldrich) was dissolved in 10 mM Tris-HCl (pH 8) to prepare a 2% stock solution and was added to the incubation media as a 20× solution. Forty micromolar resorufin, pentoxyresorufin (PR), and methoxyresorufin (MR) (S1 Fig) (AnaSpec, USA) were dissolved in DMSO to prepare 100× solutions and were added to the final concentrations 0.1, 0.5, and 5 μM, respectively. The controls received an equivalent amount of the vehicle (DMSO). The incubation assays for each xenobiotic concentration and each developmental stage of the parasite were conducted in triplicate. Ethylenediaminetetraacetate (EDTA) (anticoagulant)-treated blood and bile were isolated from a hamster and were diluted 4-fold for the experiments.

### Primer design

Primers for real-time PCR were designed using the transcriptome of maritae of *O*. *felineus* from Solexa. The primers and probes were as follows: the *Ub* (ubiquitin) gene (GenBank ID: JK649790) (UB_F: 5’-TCCGCCACTCCGTCTTACGC, UB_R: 5’-ACTAGCCGATGACATGCGGTGGA) and *MrpL16* (mitochondrial ribosomal protein L16) gene (GenBank ID: JK649791) (MrpL16_F: 5’-TCCCTTCCCGGCTCGTTTCGT, MrpL16_R: 5’-AGTGCTTGGCGAGCATCAGCA, MrpL16 Probe: 5’-R6G-ACAAGAGTTGCTGGACTGCGAGAA-BHQ2 (Synthol, Russia); paramyosin gene (GenBank ID:AF311774.1) (Paramyosin_F: 5’-AGAACGTCGCCTGCGCGAGG; Paramyosin_R: 5’-GGGCCCGATCGGCGGCTT); alfa-tubulin gene (GenBank ID: JK624299) (TUA_F: 5’-CGCGTCCGATGGTGTACCGTCC; TUA_R: 5’-GGTGCGAACCGGCACTTACCGT); *CYP* gene (GenBank ID: JF920147) (CYP_F2: 5’-ACTGGAGAATAGCAACCAAACGCCA; CYP_R1: 5’-CCCGTTCTCCATCTCGCACATCG). To measure the level of *CYP* gene expression in experiments with dsRNA, the CYP primers were as follows: CYP_F3: 5’-GCCCTTCGGCTTACCCCACA; CYP_R2: 5’-CCGCTGGACCTCTTGTAAGCCCA (the full ORF spanning positions 1053–1354). For droplet digital PCR primers and probes were as follows: *MrpL16* (mitochondrial ribosomal protein L16) gene (MrpL16_F: 5’-TCCCTTCCCGGCTCGTTTCGT, MrpL16_R: 5’-AGTGCTTGGCGAGCATCAGCA, MrpL16 Probe: 5’-R6G-ACAAGAGTTGCTGGACTGCGAGAA-BHQ2 (Synthol, Russia) and *CYP* gene (CYP_F2: 5’-ACTGGAGAATAGCAACCAAACGCCA; CYP_R1: 5’-CCCGTTCTCCATCTCGCACATCG; CYP Probe: 5’-FAM-TGCCG-ATTAT-TCGCC-GAACT-ATCTG-G–RTQ1 (Synthol, Russia)).

### Total RNA extraction, cDNA synthesis, real-time PCR, and Droplet Digital PCR

In the real-time PCR assay, we used adult worms and newly excysted metacercariae (NEM) of *O*. *felineus*. For each data point, we used five adult worms extracted from the same hamster and 400 to 500 NEM for RNA isolation. For the real-time PCR assays with dsRNA, total RNA was isolated from individual adult worms.

Total RNA for real-time PCR was isolated from flukes using the TRI reagent (Ambion, USA). Concentrations of RNA were determined using a NanoDrop spectrophotometer (ND1000, NanoDrop Technologies, USA). One microgram of total RNA was used for the synthesis of single-stranded cDNA. First-strand cDNA synthesis was performed using the RevertAid Kit (Fermentas, EU). Expression levels of the genes were measured by means of real-time PCR using the EVA Green Reagent Mix (Synthol, Russia) on a CFX96 real-time PCR system (Bio-Rad, USA). As endogenous internal controls for normalization, we chose genes *Ub* and *MrpL16* because these genes had the lowest M-value (Bio-Rad) [19, 20]. Triplicate real-time PCR reactions were conducted for each sample. After the PCRs, a dissociation curve was constructed using the melting curve program of the thermal cycler to confirm the presence of a single PCR product, which was further confirmed using gel electrophoresis. Tenfold serial dilution of standard cDNA samples was used for PCR efficiency calculations. The fold change in the target gene expression (that was normalized to the control) relative to the control was calculated from the threshold cycle values (C_t_). Data analysis was performed using the CFX96 software.

To identify satisfactory RT-PCR reference genes, we evaluated expression stability across developmental stages and among seven treatment regimens with xenobiotics. Expression stability for a particular gene is reflected by the M-value calculated as a mean standard deviation of the log-transformed expression ratio across samples for the particular gene relative to other reference genes remaining in the gene panel. The calculation was performed by stepwise exclusion of individual genes with the highest M-value from the other genes until reaching the last two genes with the smallest M value. Various investigators defined M < 1.5 as an acceptable criterion for selection of RT-PCR reference genes [22].

Mann–Whitney U test was used to determine whether the differences existed between experimental mean values. *P*-values ≤ 0.05 were considered significant (Statistica 6.0). The data are shown as mean ± SEM. Results of three independent experiments are presented.

Duplex ddPCR reaction mixes were prepared as follows. 10 μL of 2× ddPCR Master Mix (Bio-Rad, USA) and CYP and MRPL16 primers (final concentration of 300 nM) and CYP and MRPL16 probes (final concentration of 180 nM) were mixed, and cDNA template added. ddPCR workflow and data analysis were performed according to the manufacturer’s instructions.

Briefly, droplets were generated in 8-well cartridges, using the QX100 droplet generator (Bio-Rad, USA) as described. Water-in-oil emulsions were transferred to a 96-well plate and amplified. Thermal cycling conditions were: 10 min denaturation at 95°C, followed by 40 cycles of a two-step thermal profile comprising 15 s denaturation at 95°C, and 60 s annealing/extension at 100% ramp rate at 60°C. After amplification, products were denatured at 98°C for 10 minutes, then cooled to 12°C. Plates were then transferred to the QX100 droplet reader (Bio-Rad, USA). Data acquisition and analysis was performed using QuantaSoft (Bio-Rad, USA). CYP gene expression levels were quantified and values were simultaneously normalized to *MrpL16* reference gene expression. The data are shown as the normalized ratio of CYP to *MrpL16* ± SD. Each run was made in duplex. Results of three independent experiments are presented.

### Chlorzoxazone hydroxylation activity

To measure 6-OH-CLZ hydroxylation activity, we used 10 adult flukes per sample. The parasites were incubated in the RPMI medium (supplemented with a 1× antibiotic (Sigma–Aldrich) and 1% glucose) at 37°C for 24 h with 170 μg/mL CLZ in an atmosphere of 5% CO_2_. 40 μM ketoconazole was added to the medium followed by incubation for 2 h before addition of CLZ. Benzoxazole (BZ) served as an internal control and was added into samples after treatment for 24 h with CLZ. The incubation medium was centrifuged to remove eggs as described above and was mixed with one volume of ACN.

A 400-μL aliquot of a sample was mixed with 0.6 mL of a solution containing four units of beta-glucuronidase of *Helix pomatia* (Sigma–Aldrich) and 0.1 M acetic-acid buffer (pH 4.5). The samples were incubated 1 hour at 37°C and were extracted twice with 2 mL of ethyl acetate and then dried at room temperature. The samples were dissolved in 26% acetonitrile and were analyzed using high performance liquid chromatography (HPLC) on a C18 reverse-phase column (250 × 2 mm, 3 μm) at the flow rate of 0.1 mL/min. CLZ, 6-OH-CLZ, and BZ were monitored by means of absorbance at 287 nm; the retention times for 6-OH-CLZ and CLZ were 6.4 and 9.45 min, respectively. For each product, retention time of the substance and of chromatographic standards was established.

### CYP activity and *in situ* visualization

The worms were placed in Petri dishes and washed five times with sterile saline. The medium was changed to the RPMI medium containing a 1× antibiotic (Sigma–Aldrich), 1% glucose, and 5 μM PR, methoxyresorufin (MR), or benzoxyresorufin (BR). The worms were incubated at 37°C in an atmosphere of 5% CO_2_ for 18 h. Ketoconazole was added to the final concentration 40 μM to the incubation medium 2 h before addition of PR. After incubation for 18 h, the worms were washed gently three times with saline (0.9% NaCl), were fixed with 4% formaldehyde (Sigma–Aldrich) in saline for 15–20 min, and were mounted on a microscope slide with the Prolong Gold Antifade Reagent (Invitrogen, USA). The slides were examined under a microscope with DAPI, rhodamine, and fluorescein filters under an AxioImager fluorescent microscope (Zeiss) [21]. As a positive control for *in situ* visualization, we used other worms after treatment for 30 min with 0.01 μg/mL resorufin (**S2 Fig**). The images were processed in the AxioVision software (Zeiss).

### Preparation and delivery of dsRNA

The dsRNA was designed to span 646 bp of the *CYP* gene (full ORF spanning at positions 331–976). The target sequence was amplified from the plasmid using primers flanked with a T7 RNA polymerase promoter sequence, indicated in underlined italic bold faced, at the 5’end. The CYP fragment was generated using primers T7CYPF: *taatacgactcactataggg*CTGGCTCAAGGTCATGGAAT and T7CYPF: *taatacgactcactataggg*CCAGGTAAGTAAACGCCCAA. An irrelevant negative control, luciferase (LUC) dsRNA, was constructed from the plasmid pGL3 (Promega) because this sequence does not match any targets in the *O*. *felineus* genome. The plasmid pGL3 (Promega) was kindly provided by Maerova A.L., Institute of Molecular and Cellular Biology SB RAS, Novosibirsk, Russia. The fragment was amplified using primers T7LUCF: *taatacgactcactataggg*TGCGCCCGCGAACGACATTTA and T7LUCR: *taatacgactcactataggg*GCAACCGCTTCCCCGACTTCCTTA [23]. The size of the amplicons was determined using electrophoresis, and these DNA fragments were purified using standard phenol–chloroform extraction. The dsRNA was synthesized using the MEGAscript RNAi Kit (Ambion, USA). The dsRNA was precipitated with one volume of 5 M ammonium acetate and 2.5 volumes of 95% ethanol, after which, the RNA was dissolved in water. Concentrations of dsRNA were determined by spectrophotometry (ND1000, NanoDrop Technologies, USA).

After recovery from hamsters at euthanasia, the worms were rinsed in sterile 0.9% NaCl to remove residual host cell debris. The flukes were maintained in the RPMI medium containing 1× antibiotic/antimycotic (Sigma–Aldrich) and 1% glucose at 37°C, in an atmosphere of 5% CO_2_ for 18–24 h. To deliver CYP dsRNA, adult worms (18–40 per treatment group) were transferred to a cuvette (4-mm gap; Bio-Rad) in 0.1 mL electroporation buffer (RPMI-1640, 1× antibiotic/antimycotic, and 1% glucose) containing 50 μg of *CYP* dsRNA or *LUC* dsRNA. Each group of worms was subjected to square wave electroporation with a single 20-ms pulse at 125 V (Gene PulserXcell, Bio-Rad). Subsequently, the worms were maintained in the RPMI culture medium 9 (three mL of the culture medium in each well of a 12-well plate) containing 1× antibiotic/antimycotic and 1% glucose at 37°C in the atmosphere of 5% CO_2_ in the air. Similarly treated worms in electroporation buffer without the dsRNA served as controls. The worms were soaked for eight days with daily refreshment of the medium. The worms were collected for analysis on days 1, 3, 5, 6, and 8 after electroporation. Images were acquired using the inverted microscope Axiovert 40CFL equipped with Axiocam ICC3 (Zeiss). This experiment was repeated three times.

To assess the capacity of worms after the RNA interference to metabolize PR, 3 randomly-selected parasites from each treatment group five days after the knockdown were treated for 20 h with pentoxyresorufin, and images were acquired using multiple fluorescence filters. The total size of the resorufin particles in each worm was measured. The images were processed in the AxioVision software (Zeiss). The data were further analyzed in Microsoft Excel spreadsheets.

To estimate the mortality rates, the Kaplan-Meier survival curves were built using the 'survival‘ (v.2.38) R package. A 95% confidence interval was calculated (out of three independent experiments) by the log-rank test using the 'survival‘ (v.2.38) R package. Statistical difference in survival log-rank (Mantel-Haenszel) test between each pair of samples was calculated.

### Accession numbers

*Ub* (ubiquitin) gene—GenBank ID: JK649790

*MrpL16* (mitochondrial ribosomal protein L16) gene—(GenBank ID: JK649791)

*Prm* (paramyosin) gene—GenBank ID: AF311774.1

*TUA* (alfa-tubulin) gene—GenBank ID: JK624299

*CYP* (cytochrome P450) gene—(GenBank ID: JF920147)

## Results

### CYP mRNA induction and selection of an internal reference gene

The level of CYP mRNA expression was assessed using real-time PCR. As endogenous internal controls for normalization, four candidate genes were chosen: paramyosin, α-tubulin (TUA), mitochondrial ribosomal protein L16 (MrpL16), and ubiquitin-related protein (Ub).

**Fig 1:**
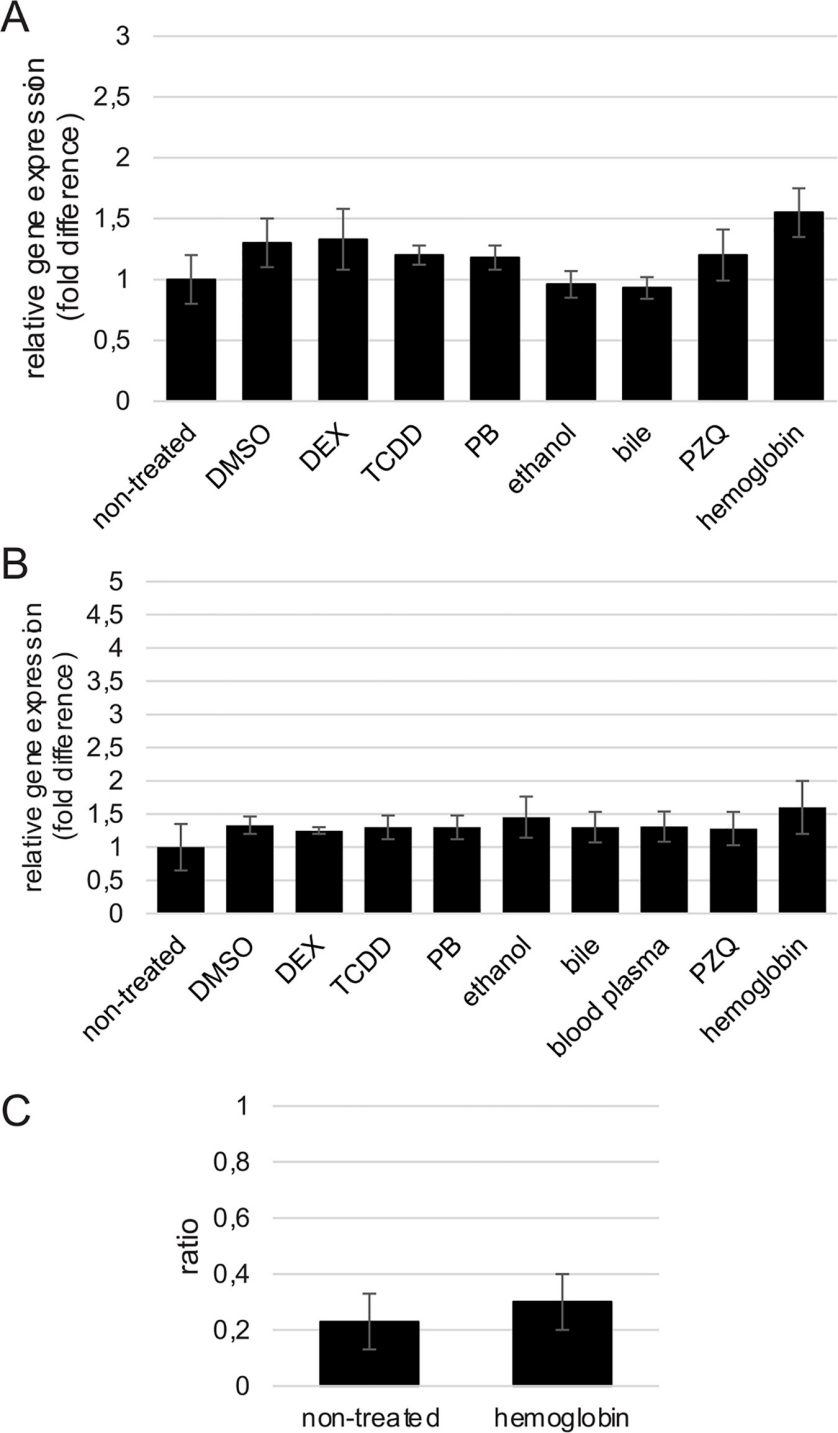
Transcriptional responses for mRNA encoding CYP following *in vitro* exposure of *Opisthorchis felineus* to xenobiotics. Analysis by real-time PCR with normalization based on the expression stability value (M-value); normalization was undertaken using *Ub* and *MrpL16* genes as endogenous internal controls (M < 1.2). Triplicate real-time PCRs were run for each sample. **A**. An adult worm; **B**. Newly excysted metacercariae (NEM). **C.** CYP mRNA level after *in vitro* treatment of *O*. *felineus* for 20 h with hemoglobin (DDPCR). CYP gene expression levels were quantified and values were simultaneously normalized to reference gene *MrpL16* expression using QuantaLife (Bio-Rad, USA). The data are shown as the normalized ratio of CYP to *MrpL16* ± S.D. Each run was made in duplex. Results of three independent experiments are presented.

Based on the outcome of preliminary experiments (see Materials and Methods section), for assessment of the CYP mRNA level, we selected two genes: Ub and MrpL16. The CYP mRNA level was measured using simultaneous normalization to these two genes. The level of CYP mRNA was 10-fold lower at the NEM stage than in adult worms. We tested the main types of inducers of CYP on adult worms and on NEM: DMSO (dimethyl sulfoxide) and TCDD (typical aryl-hydrocarbon receptor ligands), phenobarbital (constitutive androstane receptor activator), dexamethasone (DEX, a pregnane X receptor ligand), ethanol. In addition we used potential endogenous inducers: blood plasma [EDTA-treated blood] and bile from *Mesocricetus auratus* and hemoglobin (Sigma). Results of three independent experiments are presented (**Fig 1**). Treatment of adult worms and NEM for 4 h (**S3 Fig**) and for 20 h (**Fig 1A and 1B**) with any of the inducers did not change the CYP mRNA level.

Additionally, we decided to use a quantitative droplet digital PCR assay to confirm the data that hemoglobin does not induce CYP mRNA expression. There was no difference between the CYP and *MrpL16* duplex and CYP and *MrpL16* singleplex assays. ddPCR was set as a duplex assay. CYP gene expression levels were quantified and values were simultaneously normalized to *MrpL16* reference gene expression using QuantaLife (Bio-Rad, USA). The data are shown as the normalized ratio of CYP to *MrpL16* ± SD. Each run was carried out in duplex. Results of three independent experiments are presented. No difference in CYP gene expression after hemoglobin treatment of adult worms for 20 hours was evident. So, none of the compounds tested, including hemoglobin, had any effect on the expression of the P450 mRNA (**Fig 1A, 1B and 1C**)

### CYP activity

During isolation of the microsomal fraction of *O*. *felineus* proteins, CYP is isolated in the inactive state P420 [19]. Therefore, it was not possible to determine the monooxygenase activity using the method that is widely used for studies of the activity of microsomal enzymes of mammals. Previously, genes encoding other types of monooxygenases have not been observed in datasets of nucleotide sequences of *O*. *felineus*, *S*. *mansoni*, *C*. *sinensis*, *O*. *viverrini* and other parasitic species. Thus, the main enzyme that is responsible for the monooxygenase activity in *O*. *felineus* seems to be the cytochrome Р450. We hypothesized that the ability of the parasite to metabolize certain substrates of mammalian cytochromes P450 would mean functional activity of the cytochrome P450 of the parasite. It is believed that passive xenobiotic transfer through the external helminth surface is the predominant entry mechanism for most chemicals [24]. Furthermore, by adding into the incubation medium various substrates for CYP and by quantifying them, we could determine the possible spectrum of substrate specificity of the monooxygenase.

Chlorzoxazone (CLZ), a commonly used systemic myorelaxant acting on the central nervous system [25], proved to be a highly specific substrate of CYP2E1. CLZ is readily hydroxylated to 6-OH-chlorzoxazone (6-OH-CLZ) by mammalian CYP2E1 [25].

**Fig 2:**
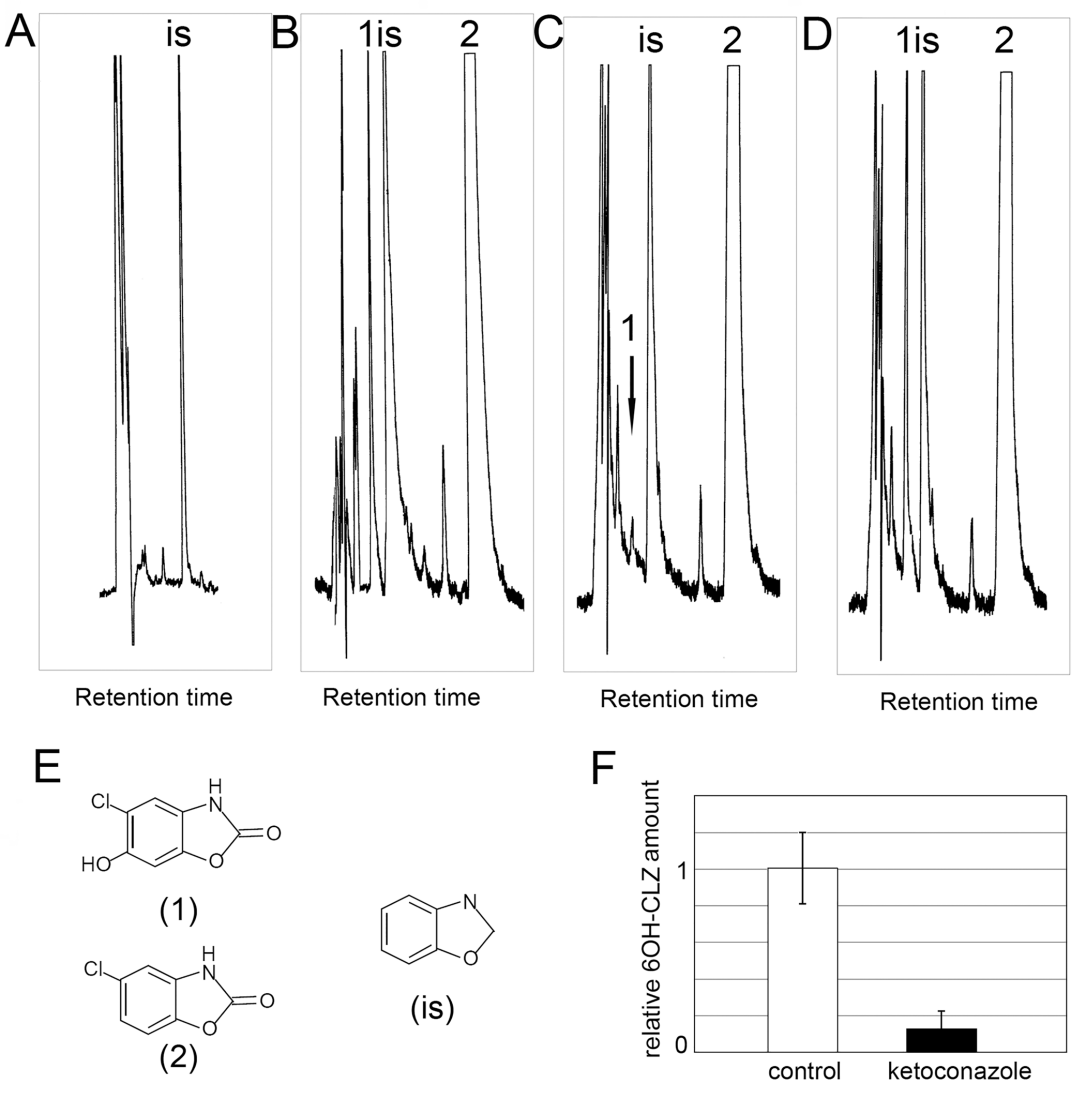
Representative chromatogram of chlorzoxazone metabolism in *Opisthorchis felineus*. (a scanned copy of the original print version). **A**. extract of *O*. *felineus* incubation media without chlorzoxazone; **B**. extract of *O*. *felineus* incubation media containing 10 mM chlorzoxazone; **C**. extract of *O*. *felineus* incubation media containing 10 mM chlorzoxazone and 40 μМ ketoconazole; A peak corresponding to retention time of 6OH-CLZ in the **2c** chromatogram is indicated with an arrow. **D**. extract **c** spiked with a standard of 6- hydroxychlorzoxazone; **E** structures of 6-OH-hydroxychlorzoxazone (**1**), chlorzoxazone (**2**), benzoxazole (**is**); **F.** Analysis of relative 6OH-CLZ amount. The area of the 6OH-CLZ peak in each chromatogram was measured. Data were expressed as a fold difference compared to the control. Data are presented as means ± S.D. Results are averaged from three independent experiments.

**Fig 2** shows a typical chromatogram of separation of the products of CLZ metabolism. In a sample of the incubation medium containing CLZ, we detected a peak corresponding to 6-OH-CLZ (**Fig 2B**). To confirm whether the metabolite formed can be produced by active CYP450, during the cultivation, we used treatment with ketoconazole. This compound is a widely known inhibitor of microsomal cytochromes. It can directly interact with different CYPs at a concentration 40nM [26, 27] and inhibit enzymatic activity of CYPs at concentration 3–40 μM [28, 29]. After simultaneous addition of ketoconazole and CLZ, the concentration of 6OH-CLZ significantly decreased and was only 10% of the 6OH-CLZ level under normal culture conditions (**Fig 2B, 2C and 2G**). To the sample of the medium during treatment with ketoconazole, we also added a standard of 6OH-CLZ: there was an increase of precisely the peak that corresponds to the retention time of 6OH-CLZ (**Fig 2D**).

### *In situ* activity

**Fig 3:**
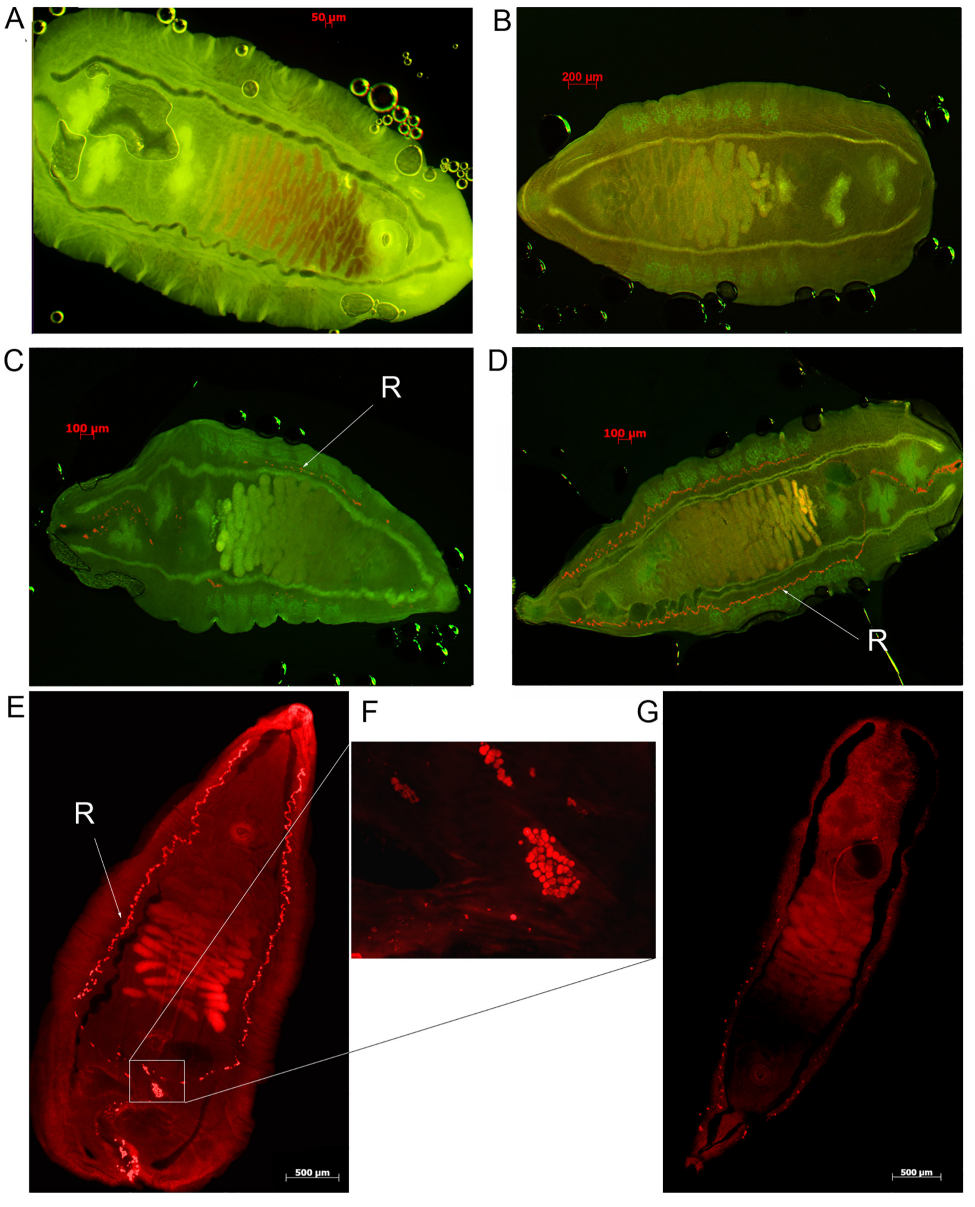
*In situ* activity of CYP. Fluorescence micrographs of *Opisthrochis felineus* after exposure *in vitro* for 20 h to several substances. **A.** A control fluke (multiple fluorescence filters: FITC, rhodamine, DAPI). **B.** Adult worms were treated with methoxyresorufin. **C.** Adult worms were treated with benzoxyresorufin. **D.** Adult worms were treated with pentoxyresorufin (PR). The resorufin (**R**) that was formed in fluke tissues is indicated with an arrow (FITC, rhodamine fluorescence filters). **E.** Adult worms were treated with penzoxyresorufin (rhodamine). **F.** Excretory granules of the resorufin formed in *O*. *felineus* treated with pentoxyresorufin. Monochrome images were acquired using a rhodamine filter (5F is a part of image 5E). **G.** A worm was treated with PR and ketoconazole (rhodamine filter).

Alkoxyresorufins PR, MR, and benzoxyresorufin (BR) are fluorogenic substrates of cytochrome P450 that yield a fluorescent product (resorufin) after enzymatic cleavage of the alkyl group (a monooxygenase dealkylation reaction) [30]. We hypothesized that an active monooxygenase in *O*. *felineus* tissues would produce a fluorescent product, which would be visible under a rhodamine filter. After 20 h of incubation with PR, large aggregates of fluorescent particles were visible (size ~5 μm) in the region of excretory channels and excretory bladder of the fluke (**Fig 3D, 3E and 3F**). It is noteworthy that fluorescence was not evident in the caeca and the surrounding tissues. After incubation with BR, we also observed formation of resorufin particles but in smaller amounts (**Fig 3C**), whereas after MR treatment, no particles were formed (**Fig 3B**). After joint treatment with PR and with ketoconazole, and inhibitor of microsomal CYP, the amount of aggregates of the fluorescent particles decreases substantially (**Fig 3G**). This provided additional evidence that the fluorescent substance that formed in the fluke tissues was a product of catalysis by CYP. Therefore, it is apparent that *O*. *felineus* can metabolize substrates specific to monooxygenases of the mammalian CYP2B and CYP3A families (PR and BR, respectively) [28] but does not metabolize the CYP1 substrate.

### Suppression of CYP mRNA in adult *O*. *felineus* by RNA interference

**Fig 4:**
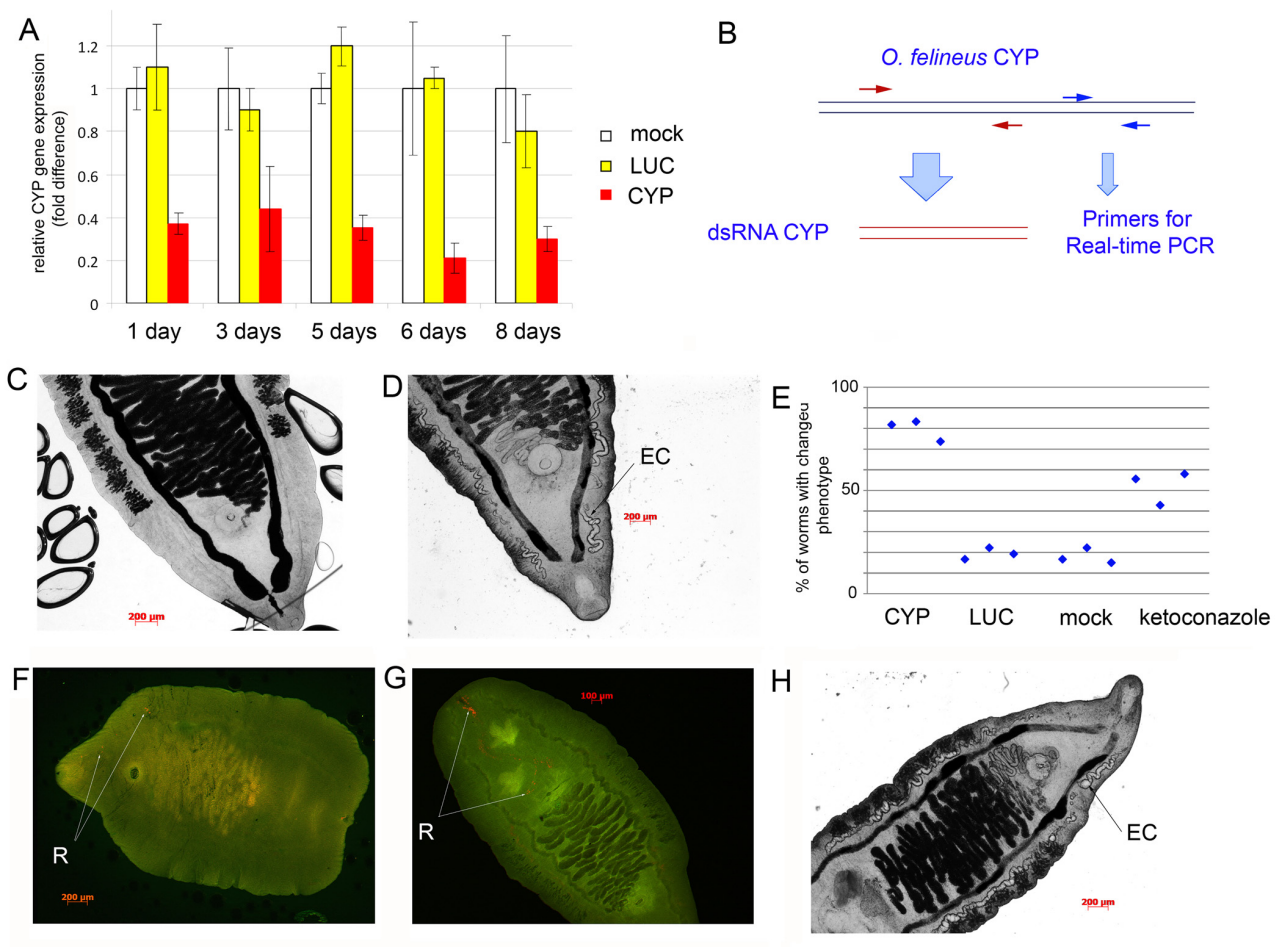
Suppression of expression of CYP in adult *Opisthorchis felineus* by RNA interference (RNAi). **A.** Transcript levels were determined using EVA-green real-time RT-PCR. The *MrpL16* gene was used for normalization. The control group (no dsRNA treatment) was compared to the negative control group transformed with *LUC* dsRNA. Three biological samples with technical duplicates were used for analysis. The data are presented as means ± SD. **B.** Primer positions for analysis of CYP gene expression. **C.** Control adult *O*. *felineus*, eight days after electroporation. EC: excretory channel, EB: excretory bladder. **D.** Worms eight days after of the knockdown of CYP mRNA. **E.** Percentage of worms with phenotypic changes in excretory system after three days of RNA interference (data of three independent experiments). Wild type–untreated worms; mock–worms subjected to electroporation without dsRNA; LUC–worms that received *LUC* dsRNA (non specific control); CYP–worms that received CYP dsRNA; keto—worms were treated with ketoconazole for three days. **F, G.** Worms five days after the knockdown of CYP (**F**) and LUC (**G**) mRNA were treated for 20 h with pentoxyresorufin, and images acquired using multiple fluorescence filters using optical sectioning by means of the AxioImager fluorescence microscope (Zeiss). The resorufin (**R**) forming in the excretory bladder is indicated with an arrow. Representative images are shown. **H.** Adult *O*. *felineus* after three days of treatment with 40 μM ketoconazole.

CYP gene expression was suppressed in *O*. *felineus in vitro* by introducing a gene-specific dsRNA using electroporation (**Fig 4B**). **Fig 4A** shows data on the relative level of CYP gene expression 1–8 days after the electroporation. The level of expression decreased by 61%, 58%, 64%, 80%, and 70%, on days 1, 3, 5, 6, and 8, respectively. The level of expression is presented in percentages of the CYP expression in control worms, which were also subjected to electroporation (with vehicle) and were kept for eight days under the same conditions (mock control). According to **Fig 4A,** treatment of the worms with the nonspecific probe LUC did not change expression of the target gene.

Worms with the suppressed expression of CYP exhibited alterations of the phenotype (**Fig 4D, S1 Dataset**). Primarily, there were changes in the shape and size of the excretory system of the worm. In particular, the size of the excretory channels and excretory bladder increased (**S1 Dataset).** These alterations started on days 3 after the gene knockdown and were maintained for eight days. Similar phenotype we also observed in other groups of worms, but the number of worms with phenotypic changes was less than number of similar worms in the CYP group (**S1 Dataset, S1 Table;** **Fig 4E**). Percentage of worms with changed phenotypes in each group is shown (**Fig 4E**).

As an additional control, to reduce the CYP protein activity, we added ketoconazole (a CYP inhibitor) to the medium with control worms. We cultured the worms for eight days under similar conditions, and the ketoconazole-containing medium was refreshed every day (**Fig 4H and 4E; S1 Dataset, S1 Table**). Changes in the phenotype of the worms were evident; the size of their excretory channels and excretory bladder was also increased (**Fig 4H**). Thus, the worms exhibited similar changes in phenotype after suppression of CYP expression and after treatment with the CYP inhibitor.

**Fig 5:**
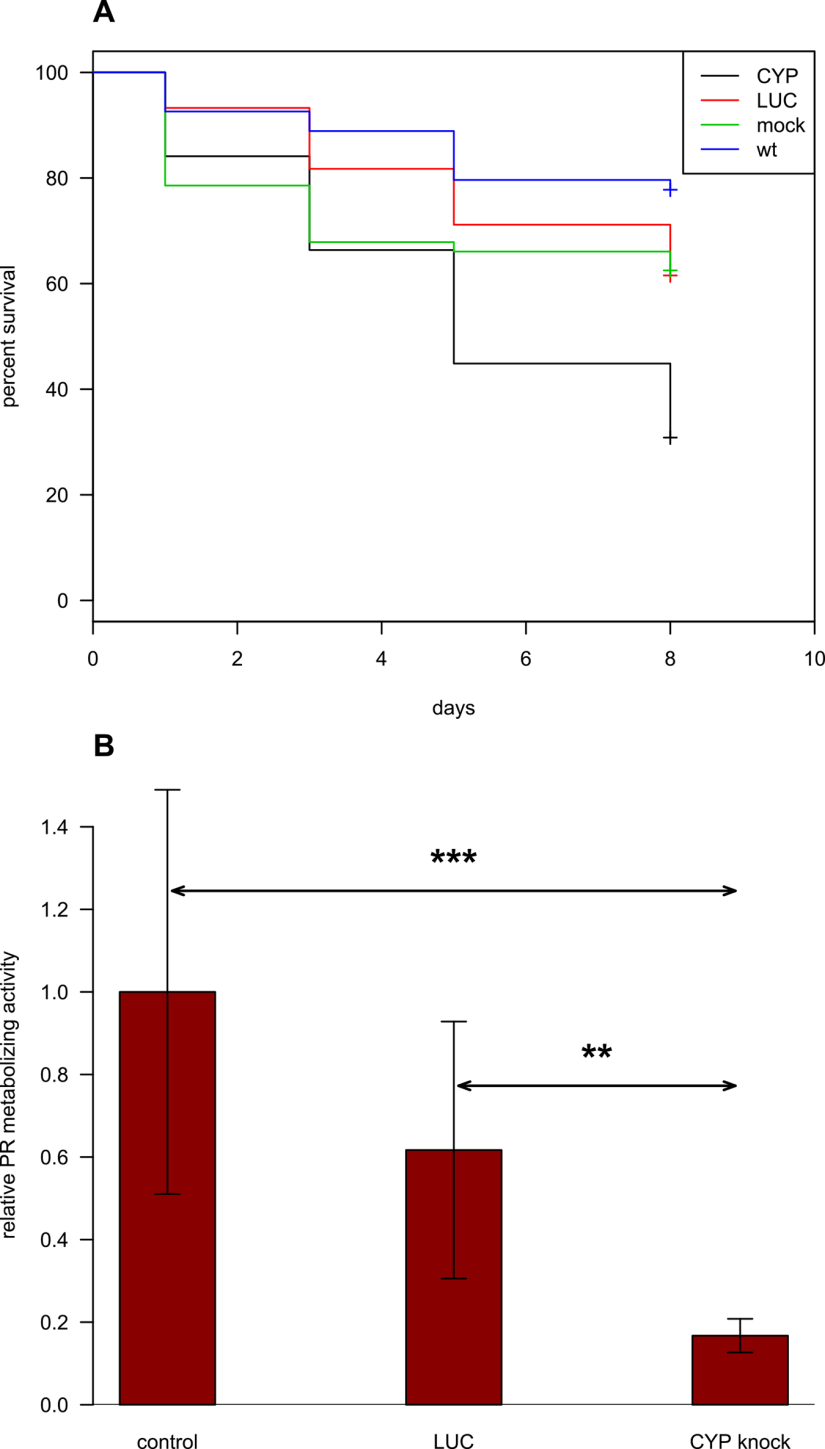
Kaplan-Meier survival curves and pentoxyresorufin metabolizing activity in worm tissues after RNA interference. **A.** A set of Kaplan-Meier survival curves (out of three independent experiments) is shown. Statistical difference in survival log-rank (Mantel-Haenszel) test between each pair of samples was calculated. The survival curves show significance when either the wild type (p<0.0001) or LUC (p<0.001) or mock control (p = 0.002) is compared to CYP group and no difference is observed when LUC group and mock control are considered (p = 0.7)('survival'(v.2.38) R package). **B.** Analysis of pentoxyresorufin (PR) metabolizing activity. Worms five days after the knockdown were treated for 20 h with pentoxyresorufin, and images were acquired using multiple fluorescence filters using optical sectioning by means of the AxioImager fluorescence microscope (Zeiss). The total size of the resorufin particles in each worm was measured. Data were expressed as a fold difference compared to the wild-type (not exposed to dsRNA) control. Data are presented as means ± S.D. Results are averaged from three independent experiments. Significance at *p* < 0.005, ***; *p* < 0.01, ** (*F*-test).

Furthermore, knockdown of the CYP gene lead to some mortality among the flukes. The worms are considered dead when all evidence of motility, including gut peristalsis, had ceased and worms had a dark colour. Eight days after the gene knockdown, viability of worms in the CYP knockdown group was 30% versus 62–79% in the control groups (**Fig 5A, S1 Table, S2 Dataset**). A 95% confidence interval was calculated (out of three independent experiments) by the log-rank test using the 'survival‘ (v.2.38) R package (**S2 Dataset**). There was no significant difference between the survival curves of worms unexposed to dsRNA (mock control) and worms exposed to LUC dsRNA. Each of the survival curves of the three control groups was significantly different to survival data obtained from worms exposed to CYP dsRNA (**Fig 5A, S2 Dataset**).

To test the hypothesis that CYP suppression reduced survival in a medium similar to the situation with the biliary tree *in vivo*, we conducted the following experiment. Eight days after CYP dsRNA treatment, the worms were kept for one day in RPMI medium containing hemoglobin, bile acids, and cholesterol. It turned out that the changes in the medium composition did not cause alterations of the phenotype, motility, or viability of the worms. To assess functional activity of the CYP enzyme five days after the knockdown of CYP mRNA, we tested the worms *in situ* for the ability to metabolize pentoxyresorufin (PR) in the tissues of the worms. The ability to metabolize PR into resorufin was decreased but remained. **Fig 4F and 4G** show granules of resorufin in tissues of worms. To assess the capacity of worms to metabolize PR, 3 randomly-selected parasites from each treatment group were treated for 20 h with pentoxyresorufin, and images were acquired using multiple fluorescence filters. The total size of the resorufin particles in each worm was measured. After being exported, the data were further analyzed in Microsoft Excel. The data are presented as means ± SD. *** *p*<v0.005, ** *p*<v0.01 (*F*-test), **Fig 5B**. Thus, the suppression of CYP mRNA in adult *O*. *felineus* by RNA interference led to reduction of CYP activity. The activity was significantly decreased and was only 18% of the PR metabolizing activity under control culture conditions (**Fig 5B**).

## Discussion

Here we investigated a gene from the liver fluke *Opisthorchis felineus* that encodes a member of the CYP family of enzymes that participate in biotransformation of xenobiotics. According to the present findings, this CYP enzyme can metabolize xenobiotics and, in parallel, can metabolize substrates selective for mammalian CYP2E1, CYP2B, CYP3A, though not CYP1A.

Recently numerous reports describe novel CYP, including enzymes of viral, bacterial, and nematode origin (reviewed in [1–3]). The number of genes encoding CYP (‘CYPome’) per genome ranges from a single isoform in some species of bacteria and fungi to dozens and hundreds in flowering plants. In free-living nematodes, the ‘CYPome’ contains several dozen divergent proteins [19]. All parasitic flatworms investigated to date possess only one cytochrome P450, regardless of the habitat. In particular, liver flukes of the families Opisthorchiidae and Fasciolidae, blood flukes (Schistosomatidae), and cyclophyllidean tapeworms of the family Taeniidae, which colonize the intestine, retain only a single cytochrome P450 [19].

It is possible that the mature *O*. *felineus* flukes that colonize bile ducts (where components of their habitat lie beyond the “barrier” of the enzymatic CYP system and of other types of oxygenases of the host’s liver) are virtually without need for a multicomponent monooxygenase system. In this context, two scenarios of functional and catalytic activity of the fluke CYP enzyme are most likely. In the first, this enzyme displays sufficiently broad substrate specificity to participate in metabolism of numerous xenobiotics, including anthelmintic drugs. In this case, the biological activity of the CYP system can be oriented toward the mechanisms of defense and adaptation of parasites. In the second, the function of this enzyme may be highly specialized and necessary primarily for one defined physiological process, for example, biotransformation of a key endogenous substrate.

Aggregates of fluorescent particles (size ~5 μm) accumulated in the region of excretory channels of the fluke. This finding suggested liberation of a product of the monooxygenase reaction (resorufin) in the excretory system and of activity of the CYP enzyme in this system but not in the caeca or pharynx. It appears that the function of the fluke CYP is linked to the excretory system of the parasite; possibly to metabolism and detoxification. The link of the CYP protein to the excretory system was also supported by changes in worm morphology following suppression of CYP by RNA interference (RNAi). Starting on day 3–4, a substantial increase in the size of and a change in the shape of the excretory channels and excretory bladder took place. The same morphological changes occurred following prolonged treatment with ketoconazole, an inhibitor of the CYP enzymatic activity [26–29]. After RNAi, levels of expression of CYP fell to only 30% of that of wild type worms, and the enzymatic activity as indicated by hydrolysis of pentoxyresorufin decreased significantly in tissues of the parasite. Taken together, the results demonstrated the efficacy of CYP RNA interference in attenuating the level of CYP transcription, which was reflected at the protein level by the reduction in enzyme activity *in situ*. Notably, increased mortality rates after CYP RNA knockdown indicated the importance of this enzyme.

The role of this cytochrome in the parasitic lifestyle remains unknown. The system with its broad substrate specificity and with conservative organization probably participates in critical physiological processes of *O*. *felineus* metabolism. A question arises whether this phenomenon is related to the synthesis of proinflammatory and potentially carcinogenic compounds, e.g., oxysterol-like and catechol estrogen quinone–like chemicals released by the flukes [8].

CYP gene exhibited constitutive expression in adult worms and in NEM. This regulation of CYP expression in *O*. *felineus* contrasts to the CYP gene induction mechanisms of mammals, where the enhancement of expression of subfamilies of CYP in response to xenobiotics can be several hundred–fold. Perhaps the absence of gene induction in *O*. *felineus* reflects continuous elevated expression of the CYP gene. Our data show that the level of expression of this gene in adult worms was similar to that of the housekeeping gene *MrpL16* and 10- to 12-fold higher compared to the metacercariae. Such elevated expression indicates the enzyme plays a key homeostatic role in the flukes resident in the bile ducts. Indeed, this phenomenon is likely to be related to the components of the habitat—bile and blood of the host.

### Conclusion

This is apparently the first study of CYP enzymes in parasitic flatworms. CYP enzymes participate in metabolism of xenobiotics and, in addition, can metabolize selective substrates for mammalian CYP2E1, CYP2B, CYP3A, but not CYP1A. It appears that the function of this CYP is linked to the excretory system of the fluke and possibly to metabolism and detoxification. Given the high level of the CYP gene expression, the search for the key endogenous substrate of CYP of parasitic flatworms is warranted, and can be anticipated to facilitate elucidation of the biochemical processes of this carcinogenic liver fluke and indeed flatworm parasites at large, and for understanding of the development of mechanisms of resistance to antiparasitic drugs.

Finally, the enzyme represents a promising drug target. Cytochromes P450 are a group of proteins involved in the synthesis of physiologically active compounds, in drug metabolism, and in biotransformation of xenobiotics. We identified only one CYP450 enzyme in *O*. *felineus*. Both from the point of view of identifying drug targets and understanding the physiology of the *Opisthorchis* parasite, CYP450 needs to be investigated.

## Supporting Information

S1 Fig Chemical structures of pentoxyresorufin (a); benzoxyresorufin (b); methoxyresorufin (c); resorufin (d). (PDF)

S2 Fig A positive control for in situ visualization. Worms after treatment for 30 min with 0.01 μg/mL resorufin were examined under a microscope with rhodamine, and fluorescein filters (Zeiss).
(PDF)

S3 Fig Transcriptional responses for mRNA encoding CYP. Worms were treated for 4 h with xenobiotics. Normalization was undertaken using *Ub* and *MrpL16* genes as endogenous internal controls (M < 1.2). Triplicate real-time PCRs were run for each sample. Results of three independent experiments are presented. **a**. Transcriptional responses for mRNA encoding CYP following *in vitro* exposure of adult worms to xenobiotics; **b**. Transcriptional responses for mRNA encoding CYP following *in vitro* exposure of newly excysted metacercariae to xenobiotics.
(PDF)

S1 Dataset Examples of adult worm phenotypes after suppression of CYP mRNA by RNA interference. Wild type–untreated worms; mock–worms subjected to electroporation without dsRNA; LUC–worms that received *LUC* dsRNA;CYP–worms that received CYP dsRNA; keto—worms were treated with ketoconazole for three days. Changes in the excretory system indicated with an arrow.
(PDF)

S2 Dataset Kaplan-Meier survival curves. A representative set of survival curves (out of three independent experiments) is shown. A 95% confidence interval was calculated by the log-rank test using the 'survival‘ (v.2.38) R package. **a**. CYP; **b**. mock control; **c**. LUC (non-specific control); **d**. wild type.
(PDF)

S1 Table A. Number of worms surviving after RNA interference (data of three independent experiments). B. Number of worms with phenotypic changes in excretory system after three of suppression by RNA interference (data of three independent experiments). Wild type–untreated worms; mock–worms subjected to electroporation without dsRNA; LUC–worms that received *LUC* dsRNA (non specific control); CYP–worms that received CYP dsRNA; keto—worms were treated with ketoconazole for three days. (XLS)
