# Supplementary figures and images for: Functional Analysis of the Unique Cytochrome P450 of the Liver Fluke Opisthorchis felineus
Source: PLoS Negl Trop Dis. 2015 Dec 1;9(12):e0004258. doi: 10.1371/journal.pntd.0004258 (PMC4666407; doi:10.1371/journal.pntd.0004258)

a

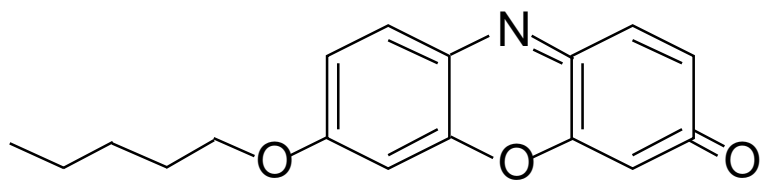

b

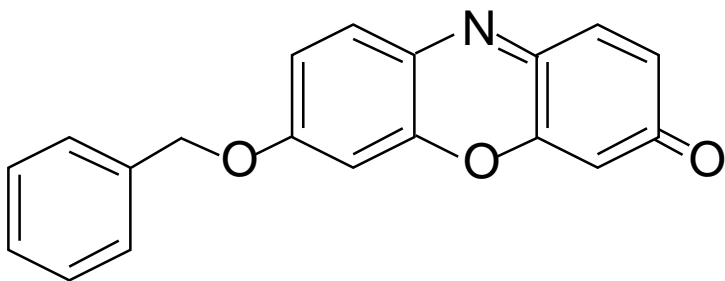

c

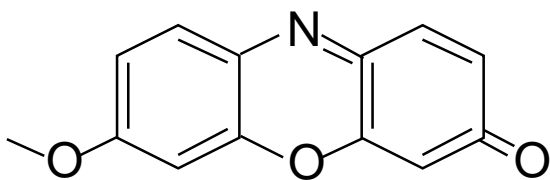

d

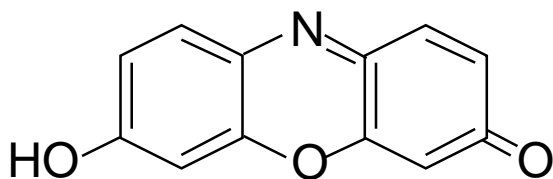

Supplement: S1 Fig — (PDF) [file pntd.0004258.s001.pdf]

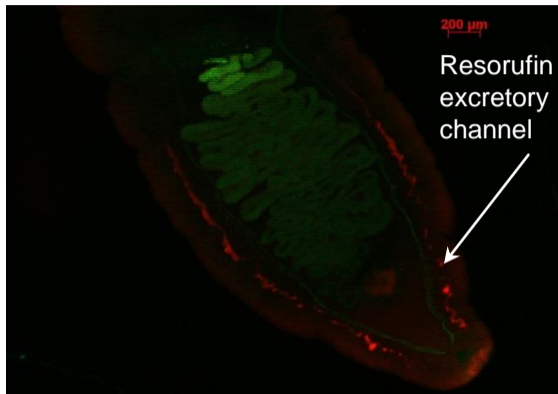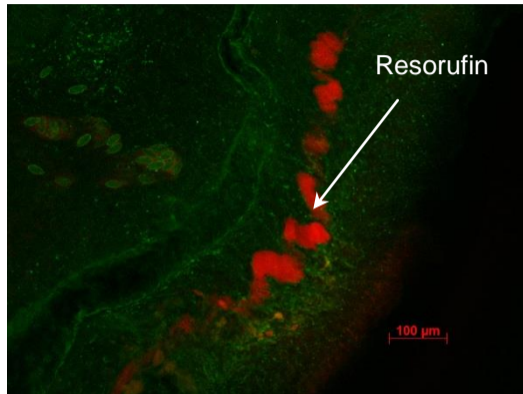

Supplement: S2 Fig — Worms after treatment for 30 min with 0.01 μg/mL resorufin were examined under a microscope with rhodamine, and fluorescein filters (Zeiss). (PDF) [file pntd.0004258.s002.pdf]

**a**

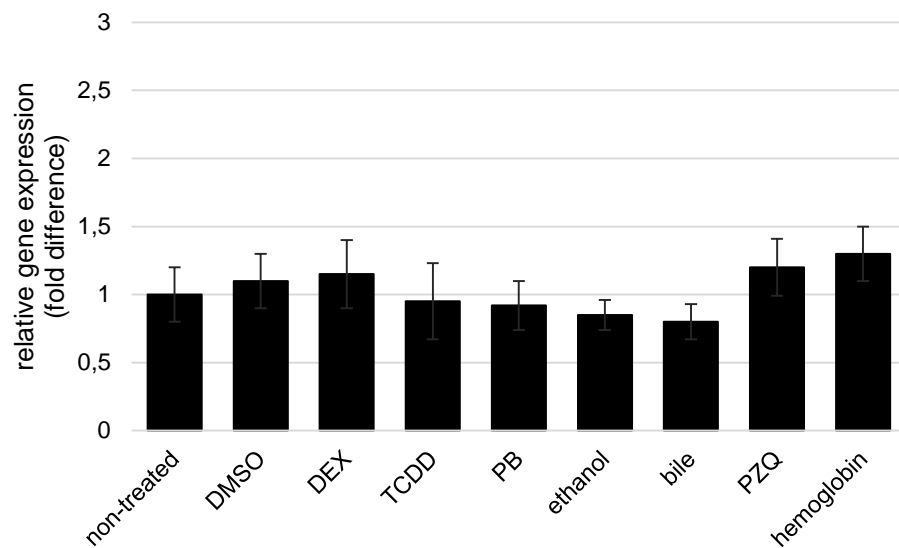

**b**

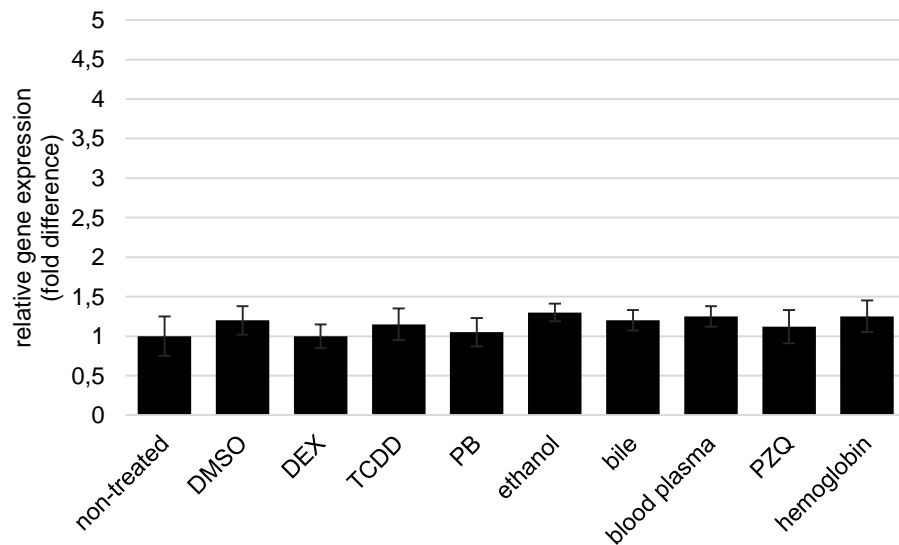

Supplement: S3 Fig — Worms were treated for 4 h with xenobiotics. Normalization was undertaken using Ub and MrpL16 genes as endogenous internal controls (M < 1.2). Triplicate real-time PCRs were run for each sample. Results of three independent experiments are presented. a. Transcriptional responses for mRNA encoding CYP following in vitro exposure of adult worms to xenobiotics; b. Transcriptional responses for mRNA encoding CYP following in vitro exposure of newly excysted metacercariae to xenobiotics. (PDF) [file pntd.0004258.s003.pdf]

control

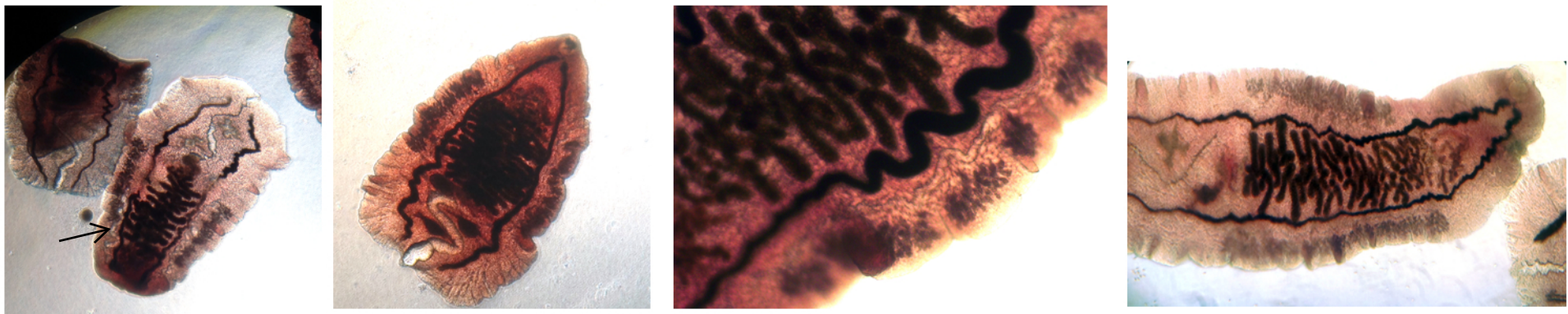

mock  
control

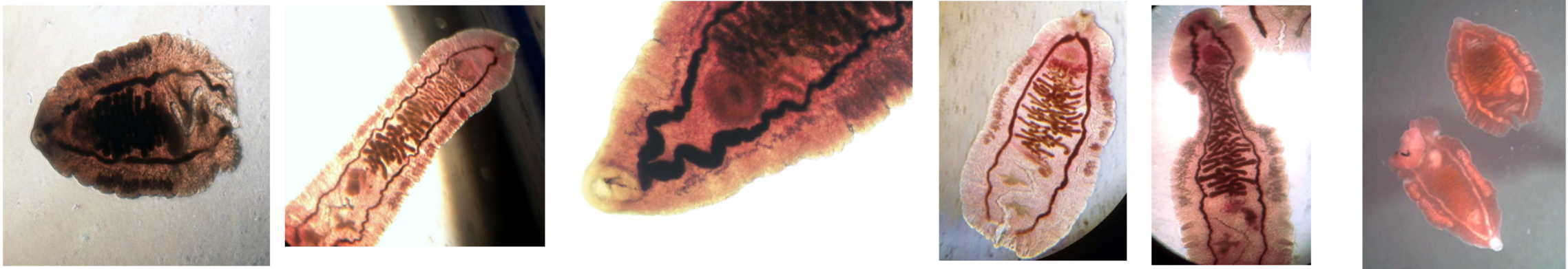

LUC  
control

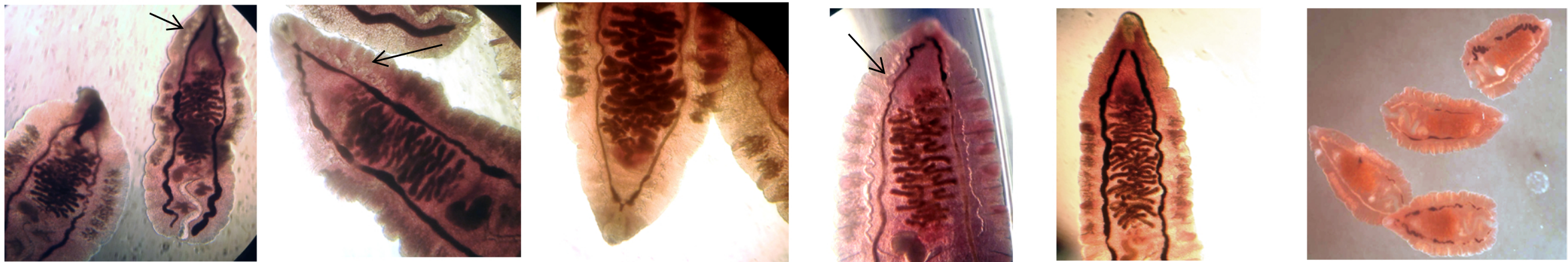

CYP

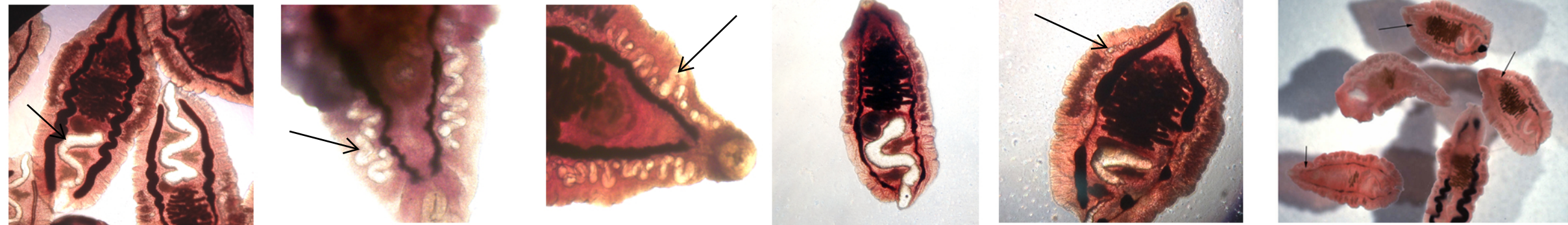

keto

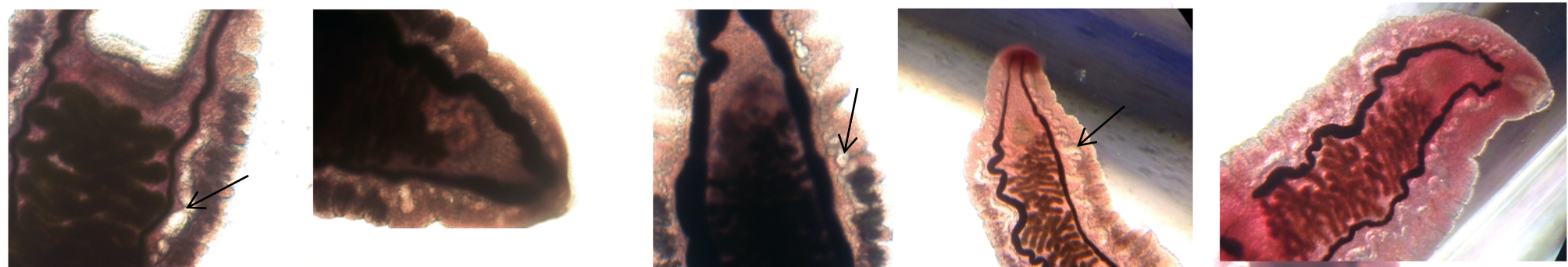

Supplement: S1 Dataset — Wild type–untreated worms; mock–worms subjected to electroporation without dsRNA; LUC–worms that received LUC dsRNA;CYP–worms that received CYP dsRNA; keto—worms were treated with ketoconazole for three days. Changes in the excretory system indicated with an arrow. (PDF) [file pntd.0004258.s004.pdf]

**a**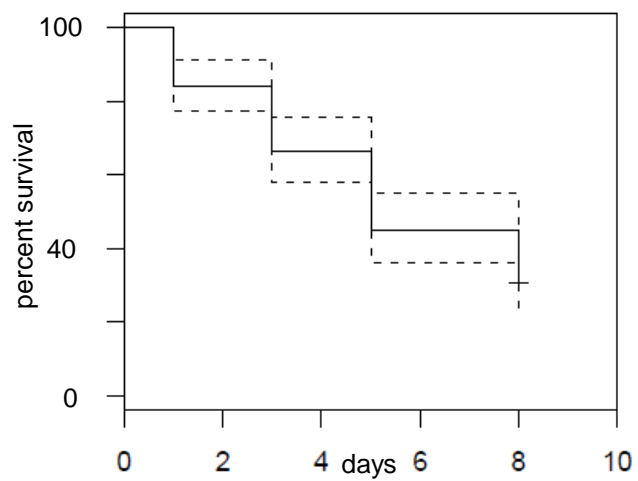**c**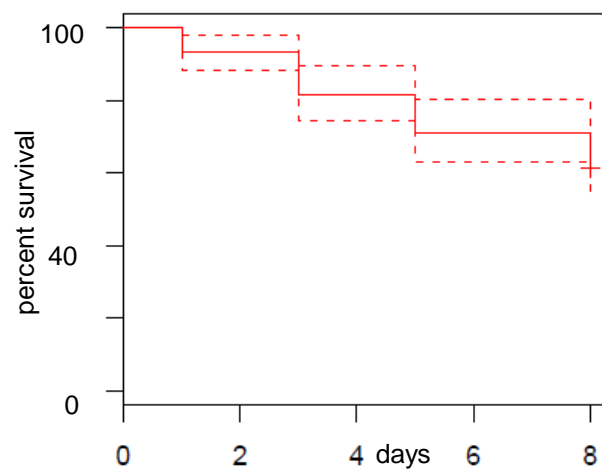**b**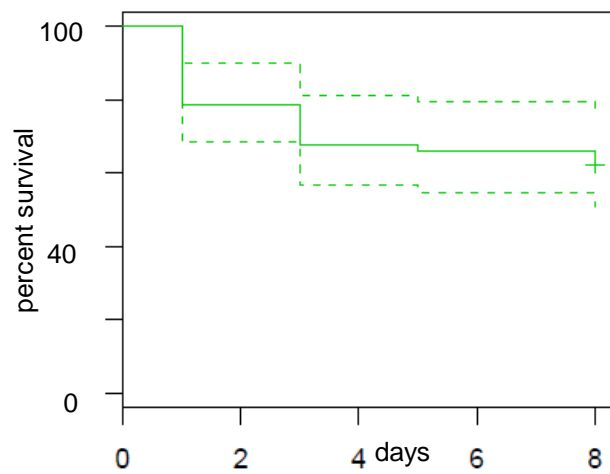**d**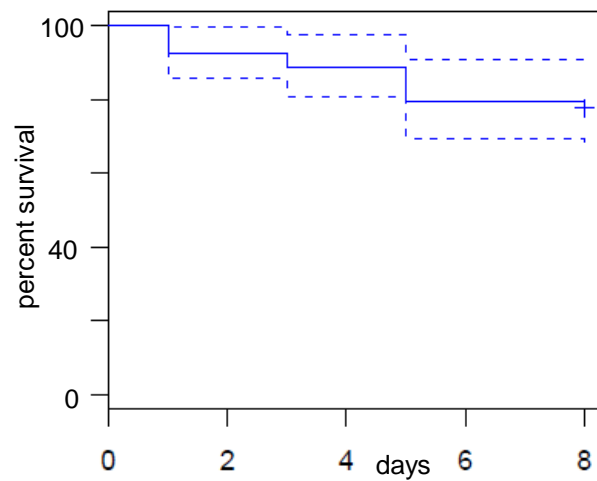

Supplement: S2 Dataset — A representative set of survival curves (out of three independent experiments) is shown. A 95% confidence interval was calculated by the log-rank test using the 'survival‘ (v.2.38) R package. a. CYP; b. mock control; c. LUC (non-specific control); d. wild type. (PDF) [file pntd.0004258.s005.pdf]
